# Moderating Effect of Age on the Relationship Between Patient Activation and Diabetes Self-Care Activities

**DOI:** 10.1093/geroni/igaa057.714

**Published:** 2020-12-16

**Authors:** Suhyun Kim

**Affiliations:** Kyungpook National University, Daegu, Republic of Korea

## Abstract

Purpose: The purpose of the study was to identify the moderating effect of age on the relationship between patient activation and diabetes self-care activities among Korean patients with type 2 diabetes mellitus. Methods: For the cross-sectional correlational study utilizing secondary data analysis, 155 patients diagnosed with type 2 diabetes were divided into two age groups: < 65 and ≥ 65 years. Original data were collected in ambulatory endocrinology units of two tertiary hospitals in South Korea from September 2016 to July 2017. Multivariate regression analyses were used, including interaction variable to detect a moderating effect of age group. Results: The level of diabetes self-care activities was similar between patients aged 65 or over and those aged less than 65 (with the possible range from 0 to 7) (3.71±1.22 vs. 3.53±1.20, t = -0.58, p = .561). Patient activation level was lower for patients aged 65 or over than those aged less than 65 (with the possible range from 0 to 100), but there was no statistical difference (61.54±4.01 vs. 68.66±16.45, t =1.68, p = .095). In multiple linear regression analysis, there was a significant interaction effect of age group (≥ 65 vs. < 65 years) and patient activation on diabetes self-care activities, controlling for the demographic and clinical variables (standardized beta = -.69, p = .021, 95% CI [-.08, -.01]). Conclusion: To encourage diabetes self-care activities, it is necessary to consider the limited effect of patient activation level for patients with type 2 diabetes aged 65 or over.

